# The Macular Carotenoids Lutein and Zeaxanthin Are Related to Increased Bone Density in Young Healthy Adults

**DOI:** 10.3390/foods6090078

**Published:** 2017-09-07

**Authors:** Emily R. Bovier, Billy R. Hammond

**Affiliations:** 1 Psychology Department, State University of New York at Oswego, Oswego, NY 13126, USA; emily.bovier@oswego.edu; 2 Behavioral and Brain Sciences, University of Georgia, Athens, GA 30602, USA

**Keywords:** carotenoids, lutein, zeaxanthin, bone, DXA

## Abstract

Lutein (L) and zeaxanthin (Z) status can be quantified by measuring their concentrations both in serum and, non-invasively, in retinal tissue. This has resulted in a unique ability to assess their role in a number of tissues ranging from cardiovascular to central nervous system tissue. Recent reports using animal models have suggested yet another role, a developmental increase in bone mass. To test this, we assessed L and Z status in 63 young healthy adults. LZ status was determined by measuring LZ in serum (using HPLC) and retina tissue (measuring macular pigment optical density, MPOD, using customized heterochromatic flicker photometry). Bone density was measured using dual-energy X-ray absorptiometry (DXA). Although serum LZ was generally not related to bone mass, MPOD was significantly related to bone density in the proximal femur and lumbar spine. In general, our results are consistent with carotenoids, specifically LZ, playing a role in optimal bone health.

## 1. Introduction

In a recent experimental study using growing mice as a model, Takeda et al. (2017) found that the dietary carotenoid, lutein (L), stimulated bone formation (increasing the density of, largely, cortical bone) by suppressing bone resorption [1]. A very similar result, also using young mice as a model, was reported by Tominari et al. (2017) who also found that L enhanced bone mineralization by suppressing osteoclastic bone resorption [2]. A direct study of the role of L on the bone health of humans, however, is limited to two studies. Wattanapenpaiboon et al. (2003) studied 205 subjects ranging from 26 to 86 years of age [3]. For premenopausal women, the higher areal bone mineral density (aBMD) of the lumbar spine was related to a greater dietary intake of L and zeaxanthin (Z) combined (*N* = 47, *r* = 0.35, *p* < 0.05). No effect, however, was found for men (*N* = 68; *r* = −0.18) or postmenopausal women (*N* = 90, *r* = 0.18). Sahni et al. (2009) did not find significant cross-sectional associations (*N* = 976) between the dietary intake of LZ and aBMD (at the femoral neck, trochanter, spine, and radial shaft) when only older subjects were assessed (mean age 75 years) [4]. Despite the absence of an effect at the baseline, a higher intake of LZ for male subjects (*N* = 193) was associated with less reduction in trochanter BMD after four years (*p* = 0.008). Both Wattanapenpaiboon et al. and Sahni et al. concluded that LZ was positively associated with bone health (despite their mixed results and the acknowledgments that serum LZ may not adequately characterize long-term dietary intake). 

If L and Z do offer protection against bone loss, as the results from the experimental animal data on young mice suggests, then it would be useful to understand the association between LZ status and bone health prior to the onset of the degeneration that is commonly seen in aging samples. Past research has included subjects in their 60s and 70s, whose bone health is likely to already reflect the consequences of oxidative stress.

To this end, we assessed a sample of younger subjects. LZ status was determined both by measuring fasting serum levels (a measure most likely reflecting acute intake) and retinal levels (a measure most likely reflecting longer term dietary strategies; [5,6]). Bone density was determined by dual-energy X-ray absorptiometry (DXA) focusing on the proximal femur and lumbar spine.

## 2. Materials and Methods

### 2.1. Subjects

A total of 63 subjects (Females, *N* = 39; Males, *N* = 24; average age of 22.5 years) were recruited from The University of Georgia and the surrounding Athens area. All subjects gave informed consent and an initial measurement of MP density at the Vision Sciences Laboratory. The initial consent process involved an explanation of all experimental procedures (visual assessments, DXA assessments, etc.). Within two to four weeks, subjects completed assessments of aBMD at the UGA Bone Clinic. At this time, the experimenter reviewed the DXA procedure (use of X-ray and effects on humans) and subjects signed an additional consent to participate (female subjects verified they were not pregnant). Finally, subjects returned to the Vision Lab for a second assessment of retinal LZ (an average over the two visits was used in all subsequent analyses). Additional assessments included a routine physical exam (for height and weight measures to assess body mass index; BMI, kg/m^2^) and a blood draw to determine serum LZ, at which time subjects completed questionnaires related to health habits. This study was reviewed and approved by the Institutional Review Board at the University of Georgia.

### 2.2. Assessment of LZ Status: Macular Pigment & Serum LZ

Retinal LZ status was assessed noninvasively by measuring the optical density of the macular pigments using a well-validated psychophysical method [7]. This technique, known as customized heterochromatic flicker photometry [8], involved measuring a subject’s spectral sensitivity at peak macular pigment absorbance (460 nm) and at a reference wavelength not absorbed by the pigments (570 nm). These wavelengths are presented in counter-phase, and the flicker rate is customized to achieve an optimal null zone for a given subject. The measurements were made at several locations within the central foveal region (7.5′, 30′, 60′, and 105′ retinal eccentricity) and at seven degrees in the periphery (assumed to be a zero point reference).

An assessment of serum LZ required the collection of blood into 10 mL lithium heparin coated vacutainers (BD) by a licensed phlebotomist. Plasma was separated by centrifugation at 1500× *g* for 20 min at 4 °C and was then distributed into light protected Eppendorf vials tubes for storage at −80 °C. The analysis of the blood was done by the analytical laboratories of DSM Nutritional Products Ltd., Kaiseraugst, Switzerland. Serum LZ levels were quantified with a normal-phase HPLC system after extraction with an n-hexane/chlorophorm 20% (*v*/*v*) mixture (see the original description in Hammond et al., 2013 [9]).

### 2.3. Assessment of Bone Mineral Density

Body composition was assessed with dual-energy X-ray absorptiometry (DXA; Delphi A, Hologic Inc., Waltham, MA, USA). The system used an X-ray generator that produced beams at two different energy levels which passed through the subject and were measured by a detector on a scanning arm located over the subject. As each beam passed through the subject, the amount of X-ray attenuation at high or low energy levels was predicated on the chemical composition through which it passed (e.g., bone or soft tissue). The unattenuated and attenuated energy levels of the high and low X-ray beams were used to determine the amount of bone and soft tissue. The precision of DXA measurements has an accuracy report of less than 1% error [10]. Two scans were completed to yield assessments of site-specific areal bone mineral density (aBMD) for the proximal femur and lumbar spine.

### 2.4. Assessment of Health Habits: Diet & Physical Activity

A brief dietary screener was constructed for the purpose of determining the general consumption of fruits and vegetables and consumption of foods rich in calcium and vitamin D. The values for the general and specific section of the dietary screener reflected an average number of servings per week of fruits/vegetables (general section) and foods rich in calcium and vitamin D (specific section). The items for the specific section of the dietary screener were selected based on the foods with the highest concentrations of calcium and vitamin D according to the US Food and Drug Administration.

Physical activity was quantified using a seven-day physical activity questionnaire that estimated the total caloric expenditure. The screener [11] assessed the amount of time spent engaging in sleep and mild, moderate, and heavy physical activities. Examples of the activities that were considered mild, moderate, or heavy were listed on the questionnaire and explained to each subject. The screener included a formula to calculate the amount of calories expended over a seven-day period based on the responses from the subject.

## 3. Results

As shown in Table 1, the average aBMD for the proximal femur and lumbar spine was 1.00 g/cm^2^ and 1.03 g/cm^2^, respectively. Means and standard deviations of LZ status (both in the retina and serum) are also listed in Table 1. The average macular pigment was 0.46 (at 30 min retinal eccentricity), and was equivalent for male and female subjects. Serum values were as follows: Serum LZ–Mean 0.25, 95% CI (0.21, 0.28); Serum L–Mean 0.19, 95% CI (0.15, 0.21); Serum Z–Mean 0.065, 95% CI (0.06, 0.07).

**Figure 1:**
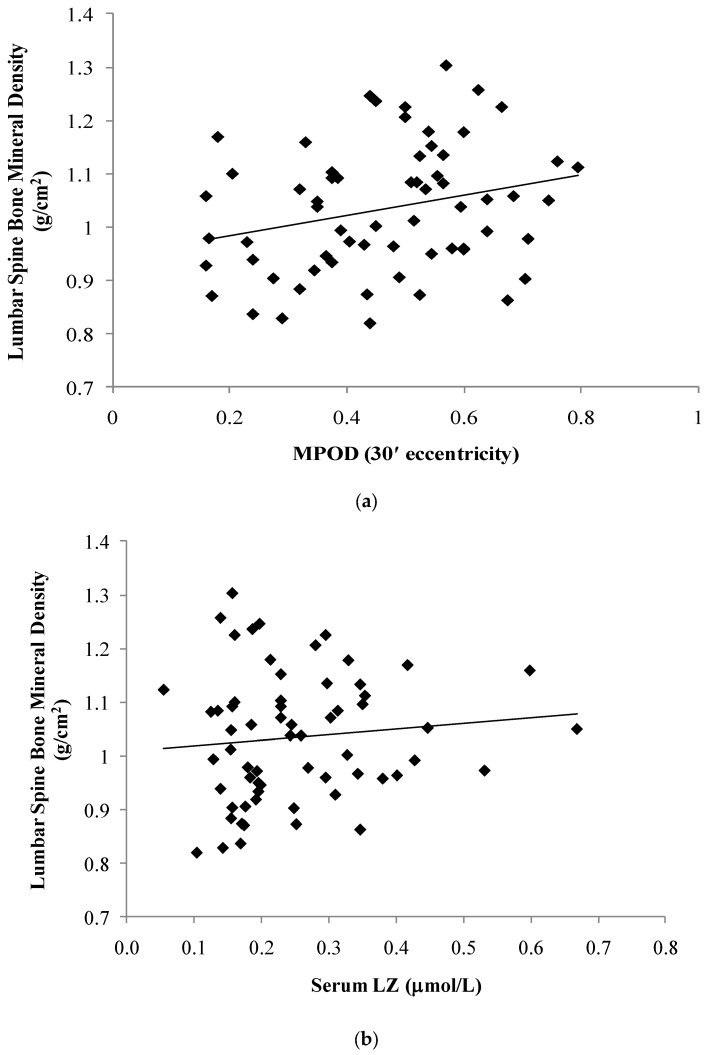
The relationship between the skeletal mass of the lumbar spine and macular pigment optical density (MPOD) at 30′ retinal eccentricity (**a**) and serum lutein/zeaxanthin (**b**).

**Figure 2:**
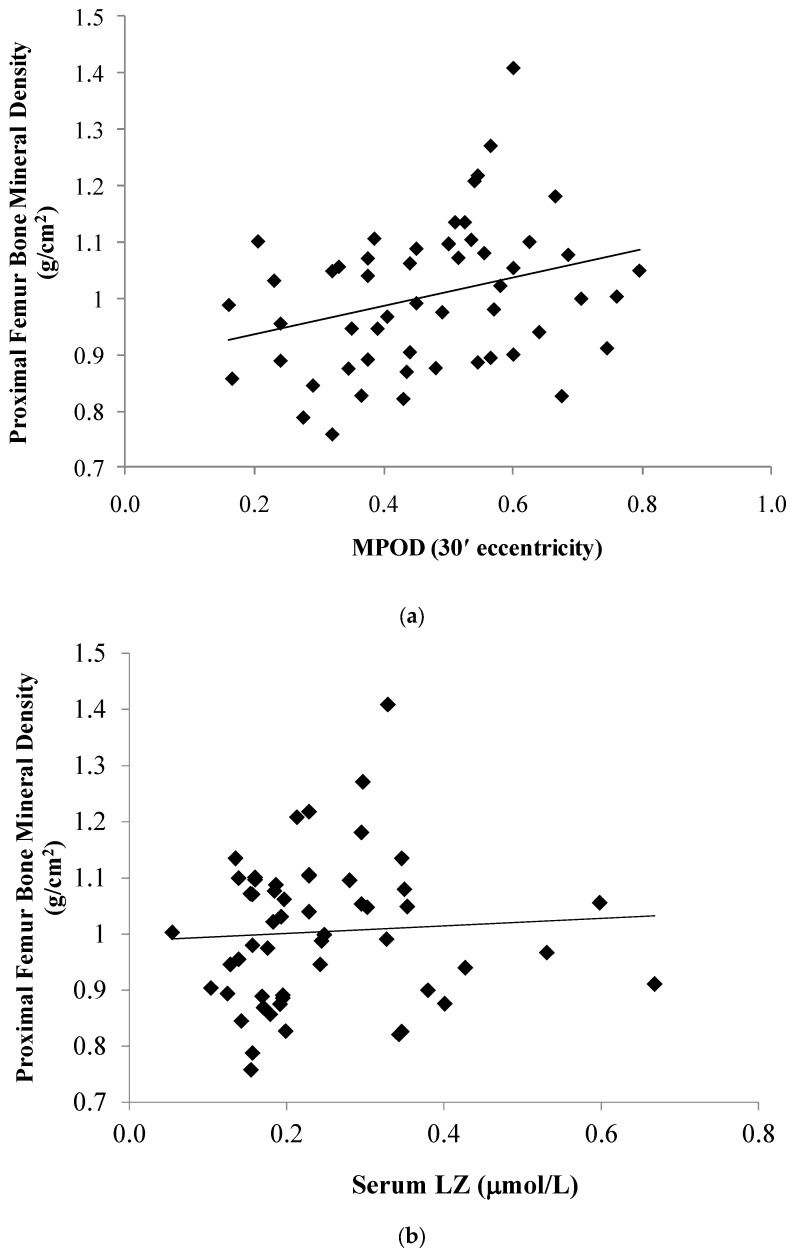
The relationship between the skeletal mass of the proximal femur and macular pigment optical density (MPOD) at 30′ retinal eccentricity (**a**) and serum lutein/zeaxanthin (**b**).

The relationship between the LZ status and aBMD of the proximal femur (PF) and lumbar spine (LS) is illustrated in Figure 1 and Figure 2. As listed in Table 2, PF and LS aBMD were positively related to MPOD at the following retinal eccentricities: 7.5′ (PF: *r* = 0.32, *p* = 0.02; LS: *r* = 0.29, *p* = 0.02); 30′ (PF: *r* = 0.30, *p* = 0.03; LS: *r* = 0.26, *p* = 0.04); and 105′ (PF: *r* = 0.43, *p* < 0.01; spine: *r* = 0.28, *p* = 0.03). Associations between aBMD and MPOD at 60′ eccentricity did not reach statistical significance (PF: *r* = 0.24, *p* = 0.08; LS: *r* = 0.22, *p* = 0.09). No significant association was found between the serum LZ and aBMD of the PF (*p* = 0.65) or LS (*p* = 0.42). A partial correlation controlling for calories expended and calcium/vitamin D intake indicated no significant association between the macular pigment (at 30 min retinal eccentricity) and bone mineral density of the proximal femur (*r* = 0.08, *p* = 0.62) and spine (*r* = 0.23, *p* = 0.17).

On average, subjects expended approximately 2574 calories per week. In addition to this marker of physical activity, we also assessed the calcium intake since these variables have been so clearly established to influence bone health. Despite our relatively rough assessment (short questionnaires), some significant relations were noted: greater physical activity was associated with a higher aBMD of the PF (*r* = 0.53, *p* < 0.01) but not the LS (*r* = 0.16, *p* = 0.28). The dietary intake (total number of servings per week) of foods rich in calcium (mean intake of approximately 19 servings) was associated with aBMD (*p* = 0.02); however, fruit and vegetable intake (mean intake of approximately seven servings per week) was not related to skeletal mass (*p* = 0.41). Body fat percentage did not account for variance in PF or LS aBMD (*p* = 0.68 and *p* = 0.30, respectively). Male and female subjects did not have significantly different PF aBMD (*p* = 0.06) or LS aBMD (*p* = 0.80).

## 4. Discussion

Our results indicate that individuals with a higher areal bone mineral density (aBMD) of the proximal femur and lumbar spine also tend to have higher MPOD. The relationship between serum LZ and skeletal mass, however, was not statistically significant. This may reflect the fact that macular pigment and bone density tend to reflect life-long habits, whereas serum LZ reflects a more short-term dietary intake. Recent experimental data are consistent with a specific role of lutein on promoting bone resorption and formation. This question of specificity is central. For example, if LZ status is simply a good marker for fruit and vegetable intake, then these correlations might simply be reflecting, e.g., other carotenoids. Other carotenoids have, in fact, been shown to promote bone health, such as lycopene [12], beta-cryptoxanthin [13], and beta-carotene [4]; although data regarding associations of these nutrients and skeletal mass are inconsistent [14,15]. It is worth noting, however, that the relations we did find were to tissue levels of LZ and not serum. Unlike serum, macular pigment density does not tend to correlate with the dietary or serum levels of other carotenoids; even the relation to LZ in serum is fairly moderate (about *r* = 0.30 for this sample). Relationships between LZ status and calories expended and calcium/vitamin D intake are listed in Table 3. In general, these markers of health habits were not related to serum LZ status. Despite macular pigment not being highly related to other food components, it was associated with a greater caloric expenditure and higher self-reported intake of calcium and vitamin D. This moderate relationship was unexpected. Food sources for carotenoids (such as green leafy vegetables) tend to be very different than food sources for calcium (such as dairy products) or vitamin D (such as fortified cereals, dark meat and fish, or sources such as the sun). No evidence currently exists showing that calcium or vitamin D (more carefully measured than in this smaller study) are significant predictors of retinal L and Z levels. Large studies that have examined the relation between MPOD, physical activity, and sun exposure have, likewise, reported no relations [16,17]. Our estimate of calcium and vitamin D intake was relatively rough and our sample size was relatively small (both factors reducing our ability to statistically control these variables). Nonetheless, given the moderate relations to MPOD, at least some level of confounding is possible and should be regarded as a limitation of this study.

In addition to effects on resorption and bone formation, if LZ is, in fact, related to a higher bone density later in life, it may be mediated by reducing oxidative stress that promotes bone loss (e.g., by helping to maintain a proper antioxidant/oxidant balance necessary for bone health [18]). A proper balance between osteoclast and osteoblast activity can be maintained with a proper balance between antioxidants and oxidants [19]. Excessive oxidative activity, however, also attenuates bone mass over time [20]. The production of reactive oxygen species (ROS) is a normal part of the bone remodeling process, which involves the coupling of osteoblasts and osteoclast functioning. Osteoclasts form and remove bone, resulting in the production of ROS, followed by an increase in bone formation by osteoblasts. However, if ROS production outweighs antioxidant mechanisms, subsequent increases in oxidative stress may result in accelerated bone loss. 

If LZ do promote bone health by lowering oxidative stress, then dietary intervention may not immediately impact bone density in young, healthy individuals who have most likely reached peak bone mass and not yet experienced significant bone loss. However, consistent with the young mouse models, dietary LZ could influence bone development in the very young. For example, do young infants with high LZ exposure have skeletal differences when compared to infants with minimal LZ exposure (e.g., infants given formula with no LZ added)? Another interesting group to study would be those reflecting not rapid development, but rapid decline (e.g., prespondylitic elderly women). Consistent with this, Sahni and colleagues (2009) were able to see an effect of LZ in the diet after following a sample of elderly subjects for four years, even though baseline associations between dietary LZ and bone density were not significant [4]. Subjects with greater LZ in their diet not only had reduced bone loss compared to other subjects, their bone density was actually higher than their baseline measurement.

## 5. Conclusions

Our results indicate a significant relationship between bone mineral density and a biomarker of LZ status that reflects long-term habits. These cross-sectional data, coupled with recent experimental data in animal models [1,2], fit well within the general conclusion [21] that maintaining a healthy diet over time can improve bone mineral status and may reduce the probability of clinical outcomes such as osteoporosis and fracture risk.

## Figures and Tables

**Table 1:** Descriptive data (mean ± standard deviation) for all subjects and stratified by sex.

	*N* = 63			Males (*N* = 24)			Females (*N* = 39)		
Macular Pigment									
MPOD 7.5′	0.57	±	0.20	0.59	±	0.20	0.57	±	0.21
MPOD 30′	0.46	±	0.16	0.47	±	0.15	0.46	±	0.17
MPOD 60′	0.30	±	0.13	0.30	±	0.11	0.30	±	0.14
MPOD 105′	0.12	±	0.08	0.13	±	0.08	0.12	±	0.08
Serum (μmol/L) *									
LZ	0.25	±	0.12	0.21	±	0.07	0.28	±	0.13
Lutein (L)	0.19	±	0.09	0.14	±	0.06	0.21	±	0.10
Zeaxanthin (Z)	0.07	±	0.03	0.06	±	0.02	0.07	±	0.03
Body Fat (percentage)									
Total	24.92	±	6.64	18.70	±	4.91	28.75	±	4.23
Arms	25.25	±	8.54	16.70	±	4.85	30.52	±	5.48
Trunk	21.60	±	6.23	17.24	±	5.88	24.28	±	4.80
Legs	29.87	±	8.62	20.85	±	5.08	35.42	±	4.76
Bone Mineral Density (g/cm^2^)									
Proximal Femur	1.00	±	0.13	1.04	±	0.15	0.97	±	0.10
Lumbar Spine	1.03	±	0.12	1.03	±	0.13	1.04	±	0.11
Health Habit Screeners									
Calories Expended	2574	±	538	2818	±	486	2440	±	525
F/V Intake (serving/week)	6.87	±	2.28	6.44	±	2.30	7.12	±	2.27
Calc/Vit D Intake (serving/week)	18.95	±	6.39	19.88	±	8.30	18.42	±	5.09
Age (years)	22.52	±	3.71	21.71	±	4.09	23.03	±	3.41
Body Mass Index (kg/m^2^)	22.87	±	2.59	23.47	±	2.33	22.51	±	2.70

* 95% Confidence intervals: Serum LZ (0.21, 0.28); Serum L (0.15, 0.21); Serum Z (0.06, 0.07). MPOD: macular pigment optical density; F/V: Fruit and vegetable.

**Table 2:** Pearson-product moment correlation coefficients and significance values (*r*|*p*) for associations between bone mineral density, LZ status, body fat, and health habits.

		Bone Mineral Density (g/cm^2^)					
		Lumbar Spine			Proximal Femur		
Macular Pigment	MPOD 7.5′	0.29	|	0.02	0.32	|	0.02
	MPOD 30′	0.26	|	0.04	0.30	|	0.03
	MPOD 60′	0.22	|	0.09	0.24	|	0.08
	MPOD 105′	0.28	|	0.03	0.43	|	0.00
Serum (mol/L)	LZ	0.10	|	0.42	0.06	|	0.65
	Lutein (L)	0.09	|	0.50	0.02	|	0.88
	Zeaxanthin (Z)	0.14	|	0.27	0.18	|	0.19
Total Body Fat Percentage		0.13	|	0.30	-0.06	|	0.68
Calories Expended		0.16	|	0.28	0.53	|	0.00
F/V Intake (serving/week)		0.12	|	0.41	0.13	|	0.41
Calc/Vit D Intake (serving/week)		0.34	|	0.02	0.38	|	0.02

**Table 3:** Pearson-product moment correlation coefficients and significance values (*r*|*p*) for associations between health habits (calories expended and calcium/vitamin D intake) and LZ status (retinal and serum).

		Calories Expended			Calcium/Vitamin D Intake		
Macular Pigment	MPOD 7.5′	0.31	|	0.03	0.30	|	0.04
	MPOD 30′	0.31	|	0.03	0.30	|	0.04
	MPOD 60′	0.22	|	0.13	0.29	|	0.05
	MPOD 105′	0.32	|	0.03	0.22	|	0.15
Serum (mol/L)	LZ	0.18	|	0.23	0.12	|	0.44
	Lutein (L)	0.12	|	0.41	0.10	|	0.50
	Zeaxanthin (Z)	0.33	|	0.02	0.15	|	0.33
